# T Cell Calcium Signaling Regulation by the Co-Receptor CD5

**DOI:** 10.3390/ijms19051295

**Published:** 2018-04-26

**Authors:** Claudia M. Tellez Freitas, Deborah K. Johnson, K. Scott Weber

**Affiliations:** Department of Microbiology and Molecular Biology, Brigham Young University, Provo, UT 84604, USA; claudiamsmicrobiology@gmail.com (C.M.T.F.); deborahkj@gmail.com (D.K.J.)

**Keywords:** calcium signaling, T cell receptor (TCR), co-receptors, CD-5, PD-1, CTL-4

## Abstract

Calcium influx is critical for T cell effector function and fate. T cells are activated when T cell receptors (TCRs) engage peptides presented by antigen-presenting cells (APC), causing an increase of intracellular calcium (Ca^2+^) concentration. Co-receptors stabilize interactions between the TCR and its ligand, the peptide-major histocompatibility complex (pMHC), and enhance Ca^2+^ signaling and T cell activation. Conversely, some co-receptors can dampen Ca^2+^ signaling and inhibit T cell activation. Immune checkpoint therapies block inhibitory co-receptors, such as cytotoxic T-lymphocyte associated antigen 4 (CTLA-4) and programmed death 1 (PD-1), to increase T cell Ca^2+^ signaling and promote T cell survival. Similar to CTLA-4 and PD-1, the co-receptor CD5 has been known to act as a negative regulator of T cell activation and to alter Ca^2+^ signaling and T cell function. Though much is known about the role of CD5 in B cells, recent research has expanded our understanding of CD5 function in T cells. Here we review these recent findings and discuss how our improved understanding of CD5 Ca^2+^ signaling regulation could be useful for basic and clinical research.

## 1. Introduction

T cells are a critical component of the adaptive immune system. T cell responses are influenced by signals that modulate the effects of the T cell receptor (TCR) and peptide-major histocompatibility complex (pMHC) interaction and initiate the transcription of genes involved in cytokine production, proliferation, and differentiation [1,2,3]. T cell activation requires multiple signals. First, the TCR engages the pMHC leading to tyrosine phosphorylation of CD3 and initiation of the Ca^2+^/Calcineurin/Nuclear factor of activated T cells (NFAT) or Protein kinase C-theta (PKCθ)/Nuclear factor-κ-light chain enhancer of activated B cells (NF-κB) or Mitogen-activated protein kinase (MAP kinase)/AP-1 pathways [4,5,6]. Second, cell surface costimulatory molecules, such as co-receptor CD28, amplify TCR-pMHC complex signals and promote stronger intracellular interactions to prevent T cell anergy [7,8]. Finally, cytokines such as interleukin-12 (IL-12), interferon α (INFα), and interleukin-1 (IL-1) promote T cell proliferation, differentiation, and effector functions [6].

Co-receptors such as CD4 and CD8 interact with MHC molecules and additional co-receptors interact with surface ligands present on antigen-presenting cells (APCs) to regulate T cell homeostasis, survival, and effector functions with stimulatory or inhibitory signals [9]. Altering co-receptor levels, balance, or function dramatically affects immune responses and their dysfunction is implicated in autoimmune diseases [10]. Stimulatory co-receptors such as CD28, inducible T cell co-stimulator (ICOS), Tumor necrosis factor receptor superfamily member 9 (TNFRSF9 or 4-1BB), member of the TNR-superfamily receptor (CD134 or OX40), glucocorticoid-induced tumor necrosis factor (TNF) receptor (GITR), CD137, and CD77 promote T cell activation and protective responses [11]. Co-receptor signaling is initiated by the phosphorylation of tyrosine residues located in immunoreceptor tyrosine-based activation motifs (ITAMs) or immunoreceptor tyrosine-based inhibitory motifs (ITIMs) [7,12]. The phosphorylated tyrosines serve as docking sites for spleen tyrosine kinase (Syk) family members such as zeta-chain-associated protein kinase 10 (ZAP-70) and Syk which activate the phospholipase C γ (PLCγ), RAS, and extracellular signal-regulated kinase (ERK) pathways in addition to mobilizing intracellular Ca^2+^ stores [13]. 

One of the best described T cell co-receptors, CD28, is a stimulatory T cell surface receptor from the Ig superfamily with a single Ig variable-like domain which binds to B7-1 (CD80) and B7-2 (CD86) [2]. Ligand binding phosphorylates CD28 cytoplasmic domain tyrosine motifs such as YMNM and PYAP and initiates binding and activation of phosphatidylinositide 3 kinase (PI3K) which interacts with protein kinase B (Akt) and promotes T cell proliferation and survival [1]. CD28 also activates the NFAT pathway and mobilizes intracellular Ca^2+^ stores through association with growth factor receptor-bound protein 2 (GRB2) and the production of phosphatidylinositol 4,5-bisphosphate (PIP2), the substrate of PLCγ1, respectively [2,14]. Blocking stimulatory co-receptors suppresses T cell effector function. For example, blocking stimulatory CD28 with anti-CD28 antibodies promotes regulatory T cell function and represses activation of auto- and allo-reactive T effector cells after organ transplantation [8,15].

T cells also have inhibitory co-receptors which regulate T cell responses [8]. The best characterized are immunoglobulin (Ig) superfamily members cytotoxic T-lymphocyte-associated protein 4 (CTLA-4) and programmed cell death protein 1 (PD-1) [8,16]. CTLA-4 binds CD80 and CD86 with greater avidity than CD28, and its inhibitory role refines early phase activation signals for proliferation and cytokine production [16,17,18,19]. PD-1, another CD28/B7 family member, regulates late phase effector and memory response [20]. Inhibitory co-receptors such as CTLA-4 and PD-1, known as “immune checkpoints”, block the interaction between CD28 and its ligands altering downstream secondary T cell activation signals [19]. Therefore, blocking CTLA-4 or PD-1 promotes effector T cell function in immunosuppressive environments [19,21]. 

There are also a number of co-receptors that have differential modulatory properties. For example, CD5, a lymphocyte glycoprotein expressed on thymocytes and all mature T cells, has contradictory roles at different time points. CD5 expression is set during thymocyte development and decreases the perceived strength of TCR-pMHC signaling in naïve T cells by clustering at the TCR-pMHC complex and reducing TCR downstream signals such as the Ca^2+^ response when its cytoplasmic pseudo-ITAM domain is phosphorylated [22,23,24,25]. The CD5 cytoplasmic domain has four tyrosine residues (Y378, Y429, Y411, and Y463), and residues Y429 and Y441 are found in a YSQP-(x8)-YPAL pseudo ITAM motif while other tyrosine residues make up a pseudo-ITIM domain [23]. Phosphorylated tyrosines recruit several effector molecules and may sequester activation kinases away from the TCR complex, effectively reducing activation signaling strength [23]. Recruited proteins include Src homology-2 protein phosphatase-1 (SHP-1), Ras GTPase protein (rasGAP), CBL, casein kinase II (CK2), zeta-chain-associated protein kinase 70 (ZAP70), and PI3K which are involved in regulating both positive and negative TCR-induced responses [26,27,28]. For example, ZAP-70 phosphorylates other substrates and eventually recruits effector molecules such as PLC gamma and promotes Ca^2+^ signaling and Ras activation which stimulates the ERK pathway and leads to cellular activation [13,29]. Conversely, SHP1 inhibits Ca^2+^ signaling and PKC activation via decreased tyrosine phosphorylation of PLCγ [13,26,30,31]. Further, Y463 serves as a docking site for c-Cb1, a ubiquitin ligase, which is phosphorylated upon CD3–CD5 ligation and leads to increased ubiquitylation and lysosomal/proteasomal degradation of TCR downstream signaling effectors and CD5 itself [32]. Thus, CD5 has a mix of downstream effects that both promote and inhibit T cell activation. Curiously, recent work suggests that in contrast to its initial inhibitory nature, CD5 also co-stimulates resting and mature T cells by augmenting CD3-mediated signaling [25,33,34,35].

Ca^2+^ is an important second messenger in many cells types, including lymphocytes, and plays a key role in shaping immune responses. In naïve T cells, intracellular Ca^2+^ is maintained at low levels, but when TCR-pMHC complexes are formed, inositol triphosphate (IP3) initiates Ca^2+^ release from intracellular stores of the endoplasmic reticulum (ER) which opens the Ca^2+^ release-activated Ca^2+^ channels (CRAC) and initiates influx of extracellular Ca^2+^ through store-operated Ca^2+^ entry (SOCE) [36,37,38,39,40,41]. The resulting elevation of intracellular Ca^2+^ levels activates transcription factors involved in T cell proliferation, differentiation, and cytokine production (e.g., nuclear factor of activated cells (NFAT)) [36,37]. Thus, impaired Ca^2+^ mobilization affects T cell development, activation, differentiation, and function [42,43]. Examples of diseases with impaired Ca^2+^ signaling in T cells include systemic lupus erythematosus, type 1 diabetes mellitus, and others [44,45].

In this review, we will focus on CD5 co-receptor signaling and its functional effects on T cell activation. First, we will discuss how the inhibitory co-receptors CTLA-4 and PD-1 modulate T cell function. Then we will compare CTLA-4 and PD-1 function to CD5 function, examine recent findings that expand our understanding of the role of CD5, and assess how these findings apply to T cell Ca^2+^ signaling. Finally, we will consider CD5 Ca^2+^ signaling regulation in T cells and its potential physiological impact on immunometabolism, cell differentiation, homeostasis, and behavior. 

## 2. Roles of Negative Regulatory T Cell Co-Receptors

### 2.1. Cytotoxic T-Lymphocyte Antigen-4 (CTLA-4)

Cytotoxic T-lymphocyte antigen-4 (CTLA-4, CD152) inhibits early stages of T cell activation by recruiting inhibitory proteins such as SHP-2 and type II serine/threonine phosphatase PP2A that interfere with T cell synapse signaling [21,46,47,48]. CTLA-4 binds B7, a protein on activated APCs, with higher affinity than the stimulatory co-receptor CD28; the resulting balance between inhibitory and stimulatory signals controls T cell activation or anergy [19,49]. In naïve T cells, CTLA-4 is located in intracellular vesicles which localize at TCR binding sites following antigen recognition and intracellular Ca^2+^ mobilization [19,50]. Like CD28, CTLA-4 aggregates to the central supramolecular activation complex (cSMAC) where it then extrinsically controls activation by decreasing immunological synapse contact time [51,52,53]. This suppresses proactivation signals by activating ligands (B7-1 and B7-2) and induces the enzyme Inoleamine 2,3-dioxygenase (IDO) which impairs Ca^2+^ mobilization and suppresses T cell activation, ultimately altering IL-2 production and other effector functions in T cells [51,54,55]. CTLA-4 also stimulates production of regulatory cytokines, such as transforming growth factor beta (TGF-β), which inhibit APC presentation and T cell effector function [47,52,53]. Compared to effector T cells (T_eff_), CTLA-4 is highly expressed in regulatory T cells (T_reg_) and plays a role in maintaining T_reg_ homeostasis, proliferation, and immune responses [16,56,57]. Total or partial CTLA-4 deficiency inhibits T_reg_’s ability to control cytokine production and can cause immune dysregulation [58,59,60,61]. Thus, CTLA-4 has an important role in the T_reg_ suppressive response [60]. Additionally, CTLA-4 mutations are associated with autoimmune diseases as thoroughly reviewed by Kristiansen et al. [62].

The loss of CTLA-4 results in removal of CTLA-4 competition with CD28 for B7-1 and B7-2 and is implicated in autoimmunity and cancer [15,63]. Because CTLA-4 inhibits TCR signaling, CTLA-4 deficiency leads to T cell overactivation as measured by increased CD3ζ phosphorylation and Ca^2+^ mobilization [64]. Thus, modulating CTLA-4 signaling is an attractive target for immunotherapies that seek to boost or impair early TCR signaling for cancer and autoinflammatory diseases [65,66]. For example, Ipilimunab, an IgG1 antibody-based melanoma treatment, is a T cell potentiator that blocks CTLA-4 to stimulate T cell proliferation and stem malignant disease progression by delaying tumor progression and has been shown to significantly increase life expectancy [19,67,68]. Additionally, Tremelimumab, a noncomplement fixing IgG2 antibody, has been tested alone or in combination with other antibodies such as Durvalumab (a PD-1 inhibitor) and improves antitumor activity in patients with non-small cell lung cancer (NSCLC), melanoma, colon cancer, gastric cancer, and mesothelioma treatment [69,70,71,72,73,74]. 

### 2.2. Programmed Death 1 (PD-1)

Programmed cell death protein-1 (PD-1, CD279) is a 288-amino acid (50–55 KDa) type I transmembrane protein and a member of the B7/CD28 immunoglobulin superfamily expressed on activated T cells, B cells, and myeloid cells [19,75,76]. PD-1 has two known ligands, PD-L1 and PD-L2, which inhibit T cell activation signals [77]. Like CTLA-4, PD-1 also inhibits T cell proliferation and cytokine production (INF-γ, TNF and IL-2) but is expressed at a later phase of T cell activation [19]. PD-1 has an extracellular single immunoglobulin (Ig) superfamily domain and a cytoplasmic domain containing an ITIM and an immunoreceptor tyrosine-based switch motif (ITSM) subunit critical for PD-1 inhibitory function [78]. Upon T cell activation, PD-1 is upregulated and initiates ITIM and ITSM tyrosine interaction with SHP-2 which mediates TCR signaling inhibition by decreasing ERK phosphorylation and intracellular Ca^2+^ mobilization [79,80]. PD-1 can block the activation signaling pathways PI3K-Akt and Ras-Mek-ERK, which inhibit or regulate T cell activation [79,81]. Thus, engagement of PD-1 by its ligand affects intracellular Ca^2+^ mobilization, IL-2 and TNF-α production, supporting PD-1’s inhibitory role in TCR strength-mediated signals [82].

PD-1 signaling also affects regulatory T cell (T_reg_) homeostasis, expansion, and function [83]. T_reg_ activation and proliferation are impacted by PD-1 expression which enhances their development and function while inhibiting T effector cells [75,84]. PD-1, PD-L, and T_regs_ help terminate immune responses [85]. Thus, PD-1 deficiency results not only in increased T cell activation, but in the breakdown of tolerance and the development of autoimmunity in diseases such as multiple sclerosis and systemic lupus erythematosus [85,86,87,88,89]. PD-1 and its ligands protect tissues from autoimmune attacks by regulating T cell activation and inducing and maintaining peripheral tolerance [90,91]. Studies done in PD-1-deficient mice observed the development of lupus-like glomerulonephritis and arthritis, cardiomyopathy, autoimmune hydronephrosis, and Type I diabetes, among other ailments [92,93,94]. PD-1 protects against autoimmunity and promotes T_reg_ function. [85]. Enhancing T_reg_ response with a PD-L1 agonist shows therapeutic potential for asthma and other autoimmune disorders [85,95]. Because PD-1 specifically modulates lymphocyte function, effective FDA-approved monoclonal antibodies targeting PD-1 are clinically available (i.e., Pembrolizumab and Nivolumab) to treat advanced malignancies [20]. Not only does blocking PD-1 decrease immunotolerance of tumor cells, it also increases cytotoxic T lymphocyte antitumor activity [20].

## 3. CD5: A Contradictory Co-Receptor

### 3.1. Overview of CD5 Signaling and Ca^2+^ Mobilization in T Cells

CD5, known as Ly-1 antigen in mice or as Leu-1 in humans, is a type I transmembrane glycoprotein (67 kDa) expressed on the surface of thymocytes, mature T cells, and a subset of B cells (B-1a) [96,97]. Although CD5 was discovered over 30 years ago, it was only in the last decade that CD5 gained attention as a key T cell activation regulator [98,99]. CD5 expression is set in the thymus during positive selection and correlates with how tightly the thymocyte TCR binds to self-peptide-MHC (self-pMHC); greater TCR affinity for self-peptide leads to increased CD5 expression in double positive (DP) thymocytes [100]. In other words, DP thymocytes that receive strong activation signals through their TCR express more CD5 than those DP thymocytes that receive weak TCR signals [100]. CD5 knockout mice (CD5^−/−^) have a defective negative and positive selection process, and therefore their thymocytes are hyper-responsive to TCR stimulation with increased Ca^2+^ mobilization, proliferation, and cytokine production [23,98]. On the other hand, because of the increased TCR avidity for self-pMHC, mature T cells with high CD5 expression (CD5^hi^) (peripheral or postpositive selection T cells) respond to foreign peptide with increased survival and activation compared to mature T cells with low CD5 expression (CD5^lo^) [34,101]. Therefore, CD5 is a negative regulator of TCR signaling in the thymus and modulates mature T cell response in the periphery [23,34,100,102].

**Figure 1:**
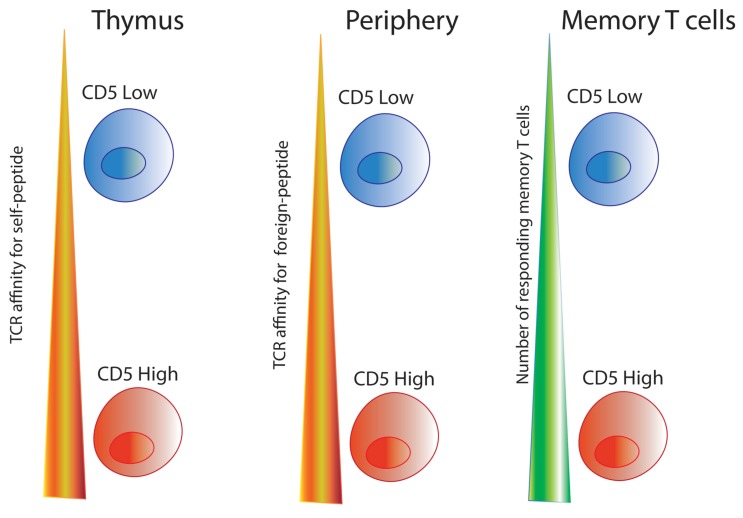
Effects of CD5 on different stages of T cell development. CD5 expression on thymocytes is directly proportional to the signaling intensity of the TCR:self-pMHC interaction. In the periphery, T cells with higher CD5 levels (CD5^hi^) are better responders to foreign-peptide. Long-lived memory cells populations are enriched for CD5^hi^ T cells [34,102,119].

While CTLA-4 and PD-1 belong to the immunoglobulin (Ig) family, CD5 belongs to group B of the scavenger receptor cysteine-rich (SRCR) superfamily and contains three extracellular SRCR domains [30,96,103]. The cytoplasmic tail of CD5 contains several tyrosine residues which mediate the negative regulatory activity independent of extracellular engagement [100,104,105]. As CD5 physically associates with TCRζ/CD3 complex upon TCR and pMHC interaction, the tyrosine residues in both TCRζ and CD5 are phosphorylated by tyrosine kinases associated with the complex [30,106,107,108,109,110]. This interaction is so intrinsic to T cell signaling that CD5 expression levels are proportional to the degree of TCRζ phosphorylation, IL-2 production capacity, and ERK phosphorylation which are critical for CD3-mediated signaling [33,111]. It is unknown whether posttranslational modifications, such as conserved domain 1 and domain 2 glycosylations, impact CD5 signaling [112,113]. CD5 is present in membrane lipids rafts of mature T cells where, upon activation, it helps augment TCR signaling, increases Ca^2+^ mobilization, and upregulates ZAP-70/LAT (linker for activation of T cells) activation [114,115,116]. This suggests that CD5 is not only a negative regulator in thymocytes, but also appears to positively influence T cell immune response to foreign antigens [117,118]. See Figure 1.

CD5 has three known ligands: CD72, a glycoprotein expressed by B cells, CD5 ligand or CD5L, an activation antigen expressed on splenocytes, and CD5 itself [120,121,122]. Crosslinking CD5L to CD5 increases intracellular Ca^2+^ concentrations [30,120,121,123,124]. Early studies with anti-CD5 monoclonal antibodies also demonstrated enhanced Ca^2+^ mobilization and proliferation, suggesting that CD5 co-stimulates and increases the T cell activation signal [125,126]. Following TCR:pMHC interaction, CD5 cytoplasmic ITAM and ITIM like-domains are phosphorylated by p56lck and bound by Src homology 2 (SH2) domain-containing protein tyrosine phosphatase (SHP-1) [108,127,128]. However, while SHP-1 affects Ca^2+^ mobilization and is a purported down-regulator of thymocyte activation, recent findings suggest that SHP-1 is not necessary for CD5 signaling as T cells deficient in SHP-1 have normal CD5 expression and continue to signal normally [26,129]. Thus, while CD5 is not a SHP-1 substrate and SHP-1 is likely unnecessary for CD5 signaling, CD5 signaling results in increased Ca^2+^ mobilization. It has yet to be resolved how CD5 can act as an inhibiting co-receptor in the thymus and as an activating co-receptor in the periphery.

### 3.2. CD5 as a Ca^2+^ Signaling Modulator

As previously mentioned, CD5 expression levels are set in the thymus during T cell development and are maintained on peripheral lymphocytes [117]. CD5 expression in T cells plays an important role during development and primes naïve T cells for responsiveness in the periphery [35,111,130]. CD5^hi^ T cells have the highest affinity for self-peptides and respond with increased cytokine production and proliferation to infection [101,131,132]. 

Our laboratory works with two TCR transgenic mouse lines with different levels of CD5 expression: LLO56 (CD5^hi^) and LLO118 (CD5^lo^) [111,117,130]. While LLO56 (CD5^hi^) and LLO118 (CD5^lo^) have similar affinity for the same immunodominant epitope (listeriolysin O amino acids 190–205 or LLO_190–205_) from *Listeria monocytogenes*, on day 7 of primary response, LLO118 (CD5^lo^) has approximately three times the number of responding cells compared to LLO56 (CD5^hi^), and conversely, on day 4 during secondary infection, LLO56 (CD5^hi^) has approximately fifteen times more cells than LLO118 (CD5^lo^) [130]. This difference is not due to differential proliferative capacity, rather LLO56 (CD5^hi^) has higher levels of apoptosis during the primary response [130]. Thus, LLO56 CD5^hi^ and LLO118 CD5^lo^’s capacity to respond to infection appears to be regulated by their CD5 expression levels [117]. LLO56 (CD5^hi^) thymocytes have greater affinity for self-peptide, which primes them to be highly apoptotic [130]. 

Recently we reported that in response to foreign peptide, LLO56 (CD5^hi^) naïve T cells have higher intracellular Ca^2+^ mobilization than LLO118 (CD5^lo^), which correlates with increased rate of apoptosis of LLO56 (CD5^hi^), as Ca^2+^ overloaded mitochondria release cytochrome c which activates caspase and nuclease enzymes, thus initiating the apoptotic pathways [35,133,134]. LLO56 (CD5^hi^) naïve T cell increased Ca^2+^ mobilization also provides additional support to the idea that CD5^hi^ T cells have an enhanced response to foreign peptide [35,134]. This supports previous research that found that upon T cell activation, increased CD5 expression is correlated with greater basal TCRζ phosphorylation, increased ERK phosphorylation, and more IL-2 production [101,111].

**Figure 2:**
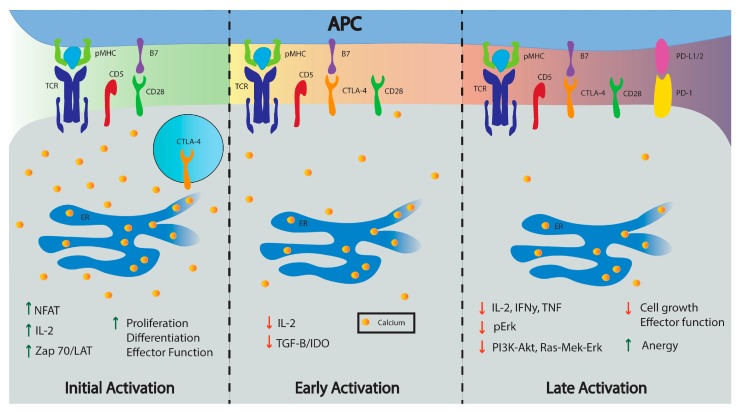
Inhibiting co-receptors modulate T cell activation by increasing (green arrows) or decreasing activity (red arrows). CD5 is present in naïve T cells and localizes to the TCR:pMHC complex during activation. Initial activation cascades signal for the release of CTLA-4 from vesicles to the cell surface while the transcription factor NFAT transcribes PD-1. CTLA-4 provides inhibitory signals during early activation while PD-1 is expressed later and inhibits later stages of T cell activation. The initial Ca^2+^ mobilization is decreased by CTLA-4 and PD-1 downstream signals. A more detailed illustration of the calcium signaling pathway (i.e., IP3, STIM 1/2, CRAC channel, calmodulin, etc.) is outlined in Figure 3.

Thus, unlike CTLA-4 and PD-1 which are expressed only on activated T cells in the periphery during early and late phases of immune response, respectively, CD5 is set during T cell development, and influences T cells both during thymic development and during postthymic immune responses [19,101,111] (see Figure 2). CD5 not only has an important inhibitory role in the thymus, but also appears to positively influence the T cell population response; for example, more CD5^hi^ T cells populate the memory T cell repertoire because CD5^hi^ naïve T cells have a stronger primary response [34,135]. CD5 finetunes the sensitivity of TCR signaling to pMHC, altering intracellular Ca^2+^ mobilization and NFAT transcription, key players in T cell effector function [19,64,126]. As Ca^2+^ signaling plays a key role in T cell activation and function, controlling Ca^2+^ mobilization in T cells through CD5 expression could influence diverse areas of clinical research including metabolism, cancer treatments, and even cognitive behavior.

## 4. Physiological Impact of CD5 Expression in T Cells

### 4.1. Metabolism

Naive T cells are in a quiescent state and rely on oxidative phosphorylation (OXPHOS) to generate ATP for survival [136,137]. Upon TCR-pMHC interaction, T cells undergo metabolic reprograming to meet energetic demands by switching from OXPHOS to glycolysis [138]. Glycolysis is a rapid source of ATP and regulates posttranscriptional production of INF-γ, a critical effector cytokine [139]. Following the immune response, most effector T cells undergo apoptosis while a subset become quiescent memory T cells. Memory T cells have lower energetic requirements and rely on OXPHOS and Fatty Acid Oxidation (FAO) to enhance mitochondrial capacity for maintenance and survival [140]. 

Ca^2+^ signaling is a key second messenger in T cell activation and Ca^2+^ ions also modulate T cell metabolism through CRAC channel activity and NFAT activation [3,141]. During TCR-pMHC binding Ca^2+^ is released from the endoplasmic reticulum (ER) where it is absorbed by the mitochondria and initiates an influx of extracellular Ca^2+^ [3]. First, the rise of cytoplasmic Ca^2+^ activates stromal interaction molecule 1 (STIM1) located on the ER membrane to interact with the CRAC channel located on the cell membrane [142]. The release of the ER store and resulting extracellular Ca^2+^ influx increases the intracellular Ca^2+^ concentration and promotes AMPK (adenosine monophosphates activated protein kinase) expression and CaMKK (calmodulin-dependent protein kinase kinase) activity [3,142,143]. AMPK senses cellular energy levels through the ratio of AMP to ATP and generates ATP by inhibiting ATP-dependent pathways and stimulating catabolic pathways [144]. This indirectly controls T cell fate as AMPK indirectly inhibits mTOR (mammalian target of rapamycin complex) [145]. Because mTOR coordinates the metabolic cues that control T cell homeostasis, it plays a critical role in T cell fate [146]. T cells that are TSC1 (Tuberous sclerosis complex 1)-deficient show metabolic alterations through increased glucose uptake and glycolytic flux [147]. 

The rise of cytoplasmic Ca^2+^ also encourages mitochondria to uptake cytoplasmic Ca^2+^ through the mitochondrial Ca^2+^ uniporter (MCU) [148]. This MCU uptake increases Ca^2+^ influx by depleting Ca^2+^ near the ER which further activates the CRAC channels and promotes STIM1 oligomerization [3,149,150,151]. Ca^2+^ uptake in the mitochondria also enhances the function of the tricarboxylic acid cycle (TAC), which generates more ATP through OXPHOS [152,153]. OXPHOS is maintained by a glycolysis product, phosphoenolpyruvate (PEP), which sustains TCR-mediated Ca^2+^-NFAT signaling by inhibiting the sarcoendoplasmic reticulum (SR) calcium transport ATPase (SERCA) pump, thus promoting T cell effector function [154,155]. Downregulation of calmodulin kinase, CaMKK2, which controls NFAT signaling, decreases glycolytic flux, glucose uptake, and lactate and citrate metabolic processes [156]. Ca^2+^ may also orchestrate the metabolic reprogramming of naïve T cells by promoting glycolysis and OXPHOS through the SOCE/calcineurin pathway which controls the expression of glucose transporters GLUT1/GLUT3 and transcriptional co-regulator proteins important for the expression of electron transport chain complexes required for mitochondria respiration [141].

**Figure 3:**
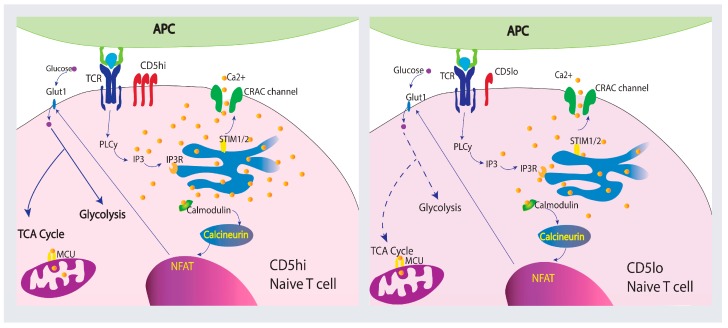
CD5 expression levels in naïve T cells may influence T cell metabolism and function. Differential levels of CD5 result in differences in Ca^2+^ mobilization in naïve T cells. CD5^hi^ naïve T cells have higher Ca^2+^ influx than CD5^lo^ naïve T cells upon TCR:pMHC interaction [35]. Ca^2+^ signaling plays a significant role in T cell activation and influences metabolism and T cell function. Differential Ca^2+^ mobilization and expression of calcineurin and NFAT affect glycolysis and mitochondrial respiration (hypothetical levels of metabolic activation are shown with dashed (low) or solid (high) arrows), suggesting CD5 expression may affect metabolic reprograming during T cell activation [141].

Co-receptor stimulation plays a pivotal role in T cell metabolism and function. A decrease in T cell Ca^2+^ signaling represses glycolysis and affects T cell effector function [152]. PD-1 and CTLA-4 depress Ca^2+^ signaling and glycolysis while promoting FAO and antibodies against CTLA-4 and PD-1 increase Ca^2+^ mobilization and glycolysis during T cell activation [157,158]. Like CTLA-4 and PD-1, CD5 modulatory function has the potential to influence T cell metabolism. Analysis of gene families modulated by CD5 in B cells found that CD5 upregulates metabolic-related genes including VEFG, Wnt signaling pathways genes, MAPK cascade genes, I-kB/NF-kB cascade genes, TGF β signaling genes, and adipogenesis process genes [159]. Therefore, proliferation differences correlated with CD5 expression in T cells may be caused by improved metabolic function as CD5^lo^ T cells seem to be more quiescent than CD5^hi^ T cells [160]. Although not much is known about how CD5 alters metabolic function in T cells, signaling strength differences of CD5^hi^ and CD5^lo^ T cell populations correlate with intracellular Ca^2+^ mobilization during activation and influence their immune response [35,111,130]. This implies that different metabolic processes may be initiated which would influence proliferation, memory cell generation, and cytokine production. Figure 3 summarizes how Ca^2+^ may be mobilized in CD5^hi^ and CD5^lo^ naïve T cells and the role Ca^2+^ may play on metabolism.

### 4.2. Neuroimmunology

The field of neuroimmunology examines the interplay between the immune system and the central nervous system (CNS) [161]. The adaptive immune system does influence the CNS as cognition is impaired by the absence of mature T cells [162]. In wild type mice, there is an increase in the number of T cells present in the meninges during the learning process, in stark contrast to mice with T helper 2 cytokine deficiencies (such as IL-4 and IL-13) who have decreased T cell recruitment and impaired learning [163]. Furthermore, regulation of T cell activation and cytokine production critically assists neuronal function and behavior, suggesting that manipulation of T cells could be a potential therapeutic target in treating neuroimmunological diseases [164,165]. 

T cells go through several microenvironments before reaching the CNS [166]. Many of the signal interactions present in these microenvironments affect T cell function and involve changes in intracellular Ca^2+^ levels [166,167]. In experimental autoimmune encephalitis (EAE), a model for human multiple sclerosis, autoreactive T cells have Ca^2+^ fluctuations throughout their journey to the CNS [166]. Prior to reaching the CNS, T cells interact with splenic stroma cells that do not display the cognate auto-antigen and this interaction produces short-lived low Ca^2+^ mobilization spikes [166]. Following entrance into the CNS, T cells encounter autoantigen-presenting cells and have sustained Ca^2+^ mobilization which results in NFAT translocation and T cell activation [166,168]. EAE mice display reduced social interaction and cognition demonstrating that autoimmune response impairs neuronal function and organismal behavior [169].

Inhibitory T cell co-receptors are implicated in CNS dysregulation and disease. Varicella zoster virus (VZV) infection is characterized by lifelong persistence in neurons. VZV increases the expression of CTLA-4 and PD-1 in infected T cells which reduces IL-2 production and increases T cell anergy [170,171]. PD-1-deficient mice (Pdcd1^−/−^) have increased T cell activation, leading to greater intracellular Ca^2+^ mobilization, and as previously discussed, increased glycolysis [86]. PD-1 deficiency causes elevated concentration of aromatic amino acids in the serum, specifically tryptophan and tyrosine, which decreases their availability in the brain where they are important for the synthesis of neurotransmitters such as dopamine and serotonin; consequently, there is an increase in anxiety-like behavior and fear in Pdcd1^−/−^ mice [86]. Therefore, increased T cell activation caused by PD-1 deficiency can affect brain function and thus, affects cognitive behavior [86].

### 4.3. Cancer

T cells are critical components of the immune response to cancer. Helper T cells directly activate killer T cells to eradicate tumors and are essential in generating a strong antitumor response alone or in concert with killer T cells by promoting killer T cell activation, infiltration, persistence, and memory formation [172,173,174,175,176,177]. Tumor-specific T cells may not mount a robust response towards cancerous cells because the tumor microenvironment has numerous immunosuppressive factors; cancerous cells also downregulate cell surface co-stimulatory and MHC proteins which suppresses T cell activation [178,179,180,181,182]. Potent antitumor immune checkpoint blockade therapies using CTLA-4 and PD-1 monoclonal antibodies augment T cell response by suppressing the co-receptors’ inhibitory signals, thereby promoting increased Ca^2+^ mobilization, glycolysis, and activation [183,184]. CTLA-4 monoclonal antibodies such as ipilimumab (Yervoy) and tremelimumab block B7-interaction and have been used to treat melanoma [47,185,186]. The monoclonal antibody pembrolizumab is highly selective for PD-1 and prevents PD-1 from engaging PD-L1 and PD-L2, thus enhancing T cell immune response [19,187,188]. Further research will address whether combining anti-CTLA-4 and anti-PD-1 antibodies will improve cancer treatments [19].

As previously mentioned, Ca^2+^ is critical for T cell activation and immune response. Manipulating Ca^2+^ signaling to enhance T cell-directed immune response against cancer is an intriguing notion, yet the means to target the Ca^2+^ response of specific cells without tampering with the metabolic processes of other cells remains elusive [189]. Antitumor activity of tumor-infiltrating lymphocytes (TIL) is inversely related to CD5 expression [99]. CD5 levels in naïve T cells are constantly tuned in the periphery by interactions with self pMHC complexes to maintain homeostasis; therefore, CD5 expression on TILs can be downregulated in response to low affinity for cancer antigens [190,191,192]. Thus, the majority of TILs are CD5^lo^ which increase their reactivity while CD5^hi^ TILs do not elicit a Ca^2+^ response and become anergic and are unable to eliminate malignant cells [99,192]. While downregulation of CD5 on TILs enhances antitumor T cell activity, CD5^lo^ T cells are also more likely to experience activation-induced cell death (AICD) as CD5 protects T cells from overstimulation [23]. To maximize TIL effectiveness, the inhibitory effects of CD5 could be blocked by neutralizing monoclonal antibodies or soluble CD5-Fc molecules combined with soluble FAS-Fc molecules to reduce the inherent AICD [23,193,194]. Soluble human CD5 (shCD5) may have a similar effect but avoids targeting issues by blocking CD5-mediated interaction via a “decoy receptor” effect. Mice constitutively expressing shCD5 had reduced melanoma and thyoma tumor cell growth and increased numbers of CD4^+^ and CD8^+^ T cells [195]. Wild type mice treated with an injection of recombinant shCD5 also had reduced tumor growth [195]. Finally, CD5-deficient mice engrafted with B16-F10 melanoma cells had slower tumor growth compared to wild type C57BL/6 mice [196]. This evidence suggests that CD5, along with PD-1 and CTLA-4, may be a potential target to specifically modulate T cell Ca^2+^ mobilization in an immunosuppressive tumor setting. 

### 4.4. Microbiome

The gut microbiome, including the bacteria and their products, forms a dynamic beneficial symbiosis with the immune system influencing host genes and cellular response. The gut microbiome shapes and directs immune responses while the immune system dictates the bacterial composition of the gut microbiome [197]. As the gut is the major symbiotic system intersecting the immune system and microbiota, understanding their connection has implications for immune system development and function as the gut microbiome is involved in protecting against pathogens, influencing states of inflammation, and even affecting cancer patient outcomes [198,199]. 

The gut microbiome primes immune responses [200]. Alteration in the microbial composition can induce changes in T cell function in infectious disease, autoimmunity, and cancer [201]. For example, mice treated with antibiotics which restrict or reduce the microbial environment exhibit impaired immune response because their T cells have altered TCR signaling and compromised intracellular Ca^2+^ mobilization in infectious disease and cystic fibrosis models [202,203,204]. In contrast, administering oral antibiotics to mice with EAE increases the frequency of CD5^+^ B cell subpopulations in distal lymphoid sites and confers disease protection [205]. In cancer, the microbiome also influences patient response to immune checkpoint inhibitors such as CTLA-4 and PD-1 [206,207]. Mice and melanoma patients immunized or populated with *Bacteriodes fragilis* respond better to treatment with Ipilimumab, a monoclonal antibody against CTLA-4 [198]. Similarly, tumor-specific immunity improved when anti-PD-1/PD-L1 monoclonal antibodies where used in the presence of *Bifidobacterium* [208]. 

Though little is known about how CD5 influences T cell interaction with the microbiome, some tantalizing details are available. As specific bacterium promotes cancer regression during CTLA-4 and PD-1 checkpoint blockades, a CD5 blockade in conjunction with bacterial selection may also improve immune response. Such studies would lead to novel immunotherapeutic treatments for cancer and autoimmune diseases.

## 5. Conclusions

CD5, widely known as an inhibitory co-receptor in the thymus, appears to modulate the signaling intensity of peripheral T cells by increasing Ca^2+^ signaling activity and efficacy of CD5^hi^ T cells. CD5 expression levels in the periphery correlates with intracellular Ca^2+^ mobilization, suggesting that CD5 promotes peripheral T cell activation and immune response. As such, CD5 may be a novel checkpoint therapy to regulate T cell activation and metabolism through altering Ca^2+^ mobilization, and could be used to affect neurological behavior, alter microbiome interactions, and treat cancer and autoinflammatory diseases. While this paper focuses on the role of co-receptor CD5 effects on calcium signaling and activation of T cells, CD5 itself may be regulated through posttranslational modifications, such as *N*-glycosylation, which may affect Ca^2+^ mobilization, T cell metabolism, activation, and function. In the future it would be interesting to determine the role of other posttranslational modifications (e.g., *N*-glycosylation, *S*-glutathionylation, lipidation) in CD5 signaling.

## Abbreviations

CTLA-4	Cytotoxic T-lymphocyte antigen 4
CD	Cluster of differenciation
PD-1	Programmed cell death protein 1
AMP	Adenosine monophosphate
ATP	Adenosine triphosphate
CaMKK	Calmodulin-dependent protein kinase kinase
AMPK	AMP-activated protein kinase
SOCE	Store-operated calcium channels
CRAC	Calcium^+^-release-activated channel
STIM	Stromal interaction molecule
SERCA	Sarcoendoplasmic reticulum calcium transport ATPase
ER	Endoplasmic reticulum
NFAT	Nuclear factor of activated T cells
INF-γ	Interferon gamma
TNF	Tumor necrosis factor
IL-2	Interleukin 2
GLUT1	Glucose transporter 1
GLUT3	Glucose transporter 3
TIL	Tumor infiltrating lymphocytes
ERK	Extracellular signal-regulated kinases
